# Association between work-related factors and health behaviour clusters among Finnish private-sector service workers

**DOI:** 10.1007/s00420-024-02069-9

**Published:** 2024-05-07

**Authors:** Elviira Lehto, Liisa Uusitalo, Tiina Saari, Ossi Rahkonen, Maijaliisa Erkkola, Jaakko Nevalainen

**Affiliations:** 1 Department of Food and Nutrition, University of Helsinki, Helsinki, Finland; 2 Faculty of Social Sciences, Work Research Centre, Tampere University, Tampere, Finland; 3 Department of Public Health, University of Helsinki, Helsinki, Finland; 4 Faculty of Social Sciences, Health Sciences, Tampere University, Tampere, Finland

**Keywords:** Workload, Working conditions, Blue-collar workers, Energy balance-related behaviours, Sleep

## Abstract

**Purpose:**

We examined how work-related factors associate with several health behaviours that appear together among the large, but less-studied, blue- and pink-collar worker group, which is characterized by low education and income levels.

**Methods:**

In 2019, we conducted a cross-sectional survey among private sector service workers (*n* = 5256) in Finland. We applied two-step cluster analysis to identify groups on the basis of leisure-time physical activity, sleep adequacy, frequency of heavy drinking, smoking status, and frequency of fruit, vegetable and berry consumption. We examined the associations with work-related factors, using multinomial regression analyses and adjusting for confounding factors.

**Results:**

We identified six clusters labelled as Moderately Healthy (28% of the participants), Healthy – Vigorous Exercise (19%), Sedentary Lifestyle (16%), Inadequate Sleep (15%), Mixed Health Behaviours (15%), and Multiple Risk Behaviours (8%). Those who perceived their work to be mentally or physically strenuous more commonly belonged to the Inadequate Sleep and Multiple Risk Behaviours clusters. Time pressure made belonging to the Inadequate Sleep, Mixed Health Behaviours, and Multiple Risk Behaviours clusters more likely. Those who were dissatisfied with their work more often belonged to the Healthy – Vigorous Exercise, Inadequate Sleep, and Multiple Risk Behaviours clusters.

**Conclusion:**

In addition of finding several considerably differing health behaviour clusters, we also found that adverse working conditions were associated with clusters characterized by multiple risk behaviours, especially inadequate sleep. Private-sector service workers’ working conditions should be improved so that they support sufficient recovery, and occupational health services should better identify co-occurring multiple risk behaviours.

**Supplementary Information:**

The online version contains supplementary material available at 10.1007/s00420-024-02069-9.

## Introduction

Across Europe, individuals with low education and income levels are more prone to smoking, eating fruit and vegetables (FV) seldom, not engaging in leisure-time physical activity (PA), and to binge drinking, although they generally consume alcohol less frequently than those with higher education or income levels (Beenackers et al. 2012; Huijts et al. 2017; Morkevicius et al. 2020; Gallus et al. 2021). One possible contributor to health behaviours is the nature of working conditions among individuals in lower socioeconomic positions. There are some indications that, for example, higher occupational PA or physically strenuous work may be associated with lower levels of leisure-time PA, although these associations have been moderate and gender specific (Mäkinen et al. 2010; Rasmussen et al. 2018) or dependent on the intensity level of PA (Gay et al. 2017). A study of Finnish municipal employees (Lallukka et al. 2004) has also demonstrated significant gender differences: a stressful and an exhaustive work life were associated with a higher likelihood of heavy drinking overall, but associations between physically strenuous work and smoking, or mentally strenuous work and heavy drinking and a healthier diet, were only found among women. Workload and exhaustion have been associated with emotional and uncontrolled eating, and exhaustion with lower levels of PA (Padilla et al. 2021). In comparison with people with permanent work contracts, those with temporary contracts have been found to have lower diet quality (Portero De La Cruz et al. 2021), but studies examining work-related factors such as working hours, physically and mentally strenuous work, and job strain (high demand/low control) have shown only weak or inconsistent associations with diet quality (Tanaka et al. 2019).

Risky health behaviours such as frequent or high alcohol consumption, smoking, low levels of PA, and an unhealthy diet often appear together (Noble et al. 2015). Although some contradictory results, mainly concerning unsafe alcohol consumption, also exist (Rebholz et al. 2012; French et al. 2018), accumulated risky health behaviour is more common among groups with inadequate income (Kino et al. 2017) or low education level (Noble et al. 2015). Moreover, alcohol consumption and smoking have accounted for a substantial proportion of the income differences in life expectancy in Nordic Countries (Östergren et al. 2019), within the last decade accompanied by other causes explaining the increased disparities in Finland (Tarkiainen et al. 2024). Few studies examining health behaviour clusters have taken sleep into account, even though both too short and long sleep duration is known to predict coronary heart disease and stroke (Cappuccio et al. 2011), and sleep disturbances increase the risk of sick leaves (Amiri and Behnezhad 2020). People with poor sleep quality and duration as well as low PA levels have also shown to have worse general health, a higher number of chronic health conditions and more psychological distress (Rayward et al. 2017). In a study conducted among truck drivers, those in a group characterized by poor sleep (short duration or sleep problems) reported more job stress and absences from work than those who were otherwise similar but had better sleep (Olson et al. 2016). Hence, accounting for inadequate sleep along with other adverse health behaviours is important, but research focusing on working conditions and health behaviour clusters is scarce. A recent study (Irizar et al. 2022) showed that those reporting high job strain were somewhat more likely to belong to a group characterized by low-risk drinking, low PA level, and insufficient FV intake.

Most of the previous studies of working conditions and health behaviours have examined separate health behaviours, been conducted among a certain occupation group, or have not separated white- and blue-collar workers in their analyses. White-collar work differs, however, from both blue-collar jobs that refer to manual labour such as maintenance services, and from pink-collar jobs that refer to person-oriented service work, often on a female-dominated branch (Lips-Wiersma et al. 2016). Service workers commonly have both low education and income level and in addition, are exposed to working conditions over which they might have little control. Thus, the novelty of our research is in examining how several work-related factors are associated with a combination of various health behaviours in this blue- and pink-collar worker group that has not previously received much attention. Due to unfavourable associations of poor sleep with health and sick leaves (Cappuccio et al. 2011; Amiri and Behnezhad 2020), we included the less-studied sleep adequacy among other health behaviours in our research. The data-driven approach allows the identification of unique patterns of leisure-time PA, sleep adequacy, smoking status, frequency of heavy drinking, and healthy eating characterising this specific worker group. We studied the association of these data-driven clusters with such work-related factors as type of industry sector, employment status, type of work contract, job satisfaction, physical and mental workload, time pressure, and being able to influence one’s work arrangements.

## Methods

The PAMEL study used data collected among private-sector service workers who were members of the Finnish Service Union United (PAM). In 2019, PAM members consisted of almost 210 000 individuals who work in the retail trade; hospitality, including restaurant, tourism, and leisure services; cleaning and property maintenance; security; and hair dressing (Service Union United 2022). The invitation to participate in the study and a link to an online questionnaire was sent to 111 850 PAM members and a link to participate in the annual PAM member survey was sent to 111 833 members. The study sample, inclusion and exclusion criteria, response rates, and content of the two questionnaires are presented in more detail in the flowchart (Appendix 1). Permission to link the survey data to the register data (information on sex, year of birth, education level, family type, and annual net income) of 2018–2019 provided by Statistics Finland was requested in the study questionnaire. Of all the members who received the invitation, 6.5% answered the study questionnaire (*n* = 6573) and the member survey (*n* = 6528). With the consent of using register data, the final sample for this study, including the cluster analyses, was 5256 participants (for participant characteristics, see Table 1). For the analyses of work-related factors, the data of both surveys, as well as the register data, were available for 3246 participants.

**Table 1:** Characteristics of PAMEL survey participants (*n* = 5256)

	Total PAMEL sample (*n* = 5256)	
	n	Mean (SD)
Age (18–73 years)	5255	43 (11.9)
Missing	1	
		%
Sex		
Woman	4163	79
Man	1092	21
Missing	1	0
Education level		
≤ Upper secondary education	3446	66
> Upper secondary education	1290	25
Missing	520	10
Marital status		
Married or registered partnership	2080	40
Cohabiting	1301	25
Not living with a partner	1875	36
Children in the household		
Yes	1948	37
No	3308	63
Self-perceived adequacy of income^1^		
Good	2918	56
Insufficient	2338	45
Self-rated health^2^		
Good	1598	30
Fairly good	2129	41
Average	1241	24
Poor	288	6

^1^ ‘Good’ combines the response options ‘fairly easily’, ‘easily’, and ‘very easily’ and ‘Insuffient’ the response options ‘with great difficulty’, ‘with difficulty’ and ‘with minor difficulty’

^2^ ‘Poor’ category combines the small category ‘poor’ with ‘fairly poor’

The PAMEL study was approved by the University of Helsinki Ethical Review Board in the humanities and social and behavioural sciences (Statement 11/2019). All the participants provided their informed written consent, and were informed that participation was voluntary and that they could withdraw from the study at any time without consequences.

### Work-related factors

The questionnaire included separate questions about the industry sector, employment status, type of work contract, mentally strenuous work, physically strenuous work, and job satisfaction (see Table 2 for response options). Being able to influence one’s work was elicited by four separate questions on the pace of work, content of tasks, division of duties between colleagues, and workplaces or workspaces.

**Table 2:** Employment status of whole PAMEL sample (*n* = 5256) and other work-related factors among those who responded to both main study survey and member survey (*n* = 3246)

	*n*	%
Job industry sector		
Retail	1354	42
Hospitality (accommodation, restaurant, and travel)	551	17
Property maintenance	335	10
Other^1^	475	15
Missing	531	16
Employment status		
Employed/partial pension	4665	89
Unemployed or laid off	591	11
Type of employment contract^2^		
Permanent, full-time	1512	47
Fixed-term/part-time	1193	37
Missing	541	17
Working under time pressure		
Very often	1053	32
Quite often	900	28
Sometimes or less often^3^	754	23
Missing	539	17
Mentally strenuous work^4^		
Fully agree	666	21
Somewhat agree	1224	38
Somewhat or fully disagree	786	24
Missing or do not know	570	18
Physically strenuous work^4^		
Fully agree	638	20
Somewhat agree	1077	33
Somewhat or fully disagree	973	30
Missing or do not know	558	17
Job satisfaction^5^		
Completely or moderately satisfied	1581	49
Equally satisfied and dissatisfied	627	19
Completely or moderately dissatisfied	505	16
Missing	533	16
Able to influence working conditions^6^		
A great deal or quite a lot	1162	36
To some extent or not at all	1506	46
Missing or do not know	578	18

^1^ Includes all small categories such as ‘safety and security’, ‘hairdressing’, and ‘other’

^2^ Type of work contract was classed as full-time or part-time employment, and as fixed-term or permanent contracts. For the analyses, we formed a new variable ‘permanent, full-time contract’ vs other type of contract

^3^ Combines the small categories ‘sometimes’, ‘rarely’, and ‘never’

^4^ For the analysis, we merged the small categories ‘somewhat disagree’ and ‘fully disagree’, and ‘cannot say’ was coded as missing

^5^ For the analysis, we merged the categories ‘completely satisfied’ and ‘moderately satisfied’ as well as ‘moderately dissatisfied’ and ‘completely dissatisfied’

^6^ If the participant chose the response options ‘a great deal’ or ‘quite a lot’ in any of the four questions, they were classified as being able to influence their work ‘a great deal or quite a lot’ and in all other cases as ‘can influence one’s work to some extent or not at all’. ‘Cannot say’ was coded as missing

### Health behaviour

*Healthy eating* was examined using two food consumption variables, one considered to be healthy and one unhealthy: (1) the sum of fruit, vegetables, and berries (FVB) and (2) the sum of sweet and savoury snacks and sugar-sweetened beverages. We elicited food consumption frequencies using a seven-point scale ranging from ‘never’ to ‘more than once a day’ and transformed it into consumption frequencies per week to enable us to calculate the sum variables.

*Smoking status* was enquired by a question on daily smoking (yes/no).

The frequency of *heavy drinking* episodes was measured on an 11-point scale, ranging from ‘daily’ to ‘never’. The original coding of heavy drinking occasions per day, week, month, or year was transformed into times per week.

*Leisure-time PA* was defined as whether leisure time was mostly spent in sedentary activities, or whether the participant engaged in moderate PA (e.g., walking or cycling), vigorous PA (e.g., running, skiing), or competitive sports. The question was a shortened version of a previously used measure (Hu et al. 2003). Few participants took part in competitive sports, hence the last two categories were merged.

*Sedentariness* during leisure time was elicited using an open-ended question as average duration per day.

*Sleep adequacy* was elicited by a three-category question on whether the participant felt that they slept sufficiently almost always, often, or rarely/almost never. The first two categories were combined for analysis to focus on those whose inadequate sleep might pose a health risk.

*Self-rated health*, used to assess the credibility of the health behaviour cluster solution, was inquired with the question “How would you describe your state of health at present?” The participants responded by using a five-point scale ranging from ‘good’ to ‘poor’.

*Sociodemographic factors* served as confounding variables if they correlated with working conditions, health behaviour cluster membership, and self-rated health. Age (at 2018) and sex were primarily retrieved and derived from the register data, and the missing values in them (*n* = 6 for both) were obtained from the questionnaire data. The highest attained level of education was recoded into a dichotomous variable of ‘upper secondary education or less’ and ‘more than upper secondary education’. Family type was derived from two separate variables, indicating marital status and children/no children in the household. Adequacy of income was measured as the self-assessed ability of the household to cover their expenses.

### Statistical methods

For descriptive purposes, we calculated the frequencies of the sociodemographic and work-related variables. Clusters were formed using two-step cluster analysis (Chiu et al. 2001), which allows clustering by continuous and categorical variables. The first step of the analysis finds a pre-clustering, which is followed by an agglomerative clustering algorithm using a log-likelihood-based distance metric to provide the final solution. First, we included all the selected health behaviour variables listed above (1–2 per each health behaviour) in the analysis. The maximum number of clusters was set at 15. We checked the predictor importance of each variable, and excluded those that had a value of < 50% of the more important variable within the same health behaviour. This left smoking, adequacy of sleep, leisure-time PA, FVB consumption, and frequency of heavy drinking in the model. The algorithm produced six clusters with good cluster quality, meaningful content, and reasonable size. To confirm this choice, we also assessed other cluster solutions by setting the number of clusters to four, five, and seven to ten. The four- and five-cluster solutions had less differentiating power than the six-cluster solution, and in the four-cluster solution, the cluster quality decreased to moderate. Cluster solutions from seven to ten, on the other hand, produced one or more very small clusters that would be difficult to analyse in terms of work-related factors. Thus, we settled with six distinctive and meaningful clusters. We labelled the largest cluster comprising 28 per cent of the participants *Moderately Healthy*. The second largest cluster we named *Healthy – Vigorous Exercise* (19%) followed by the *Sedentary Lifestyle* (16%), *Inadequate Sleep* (15%), *Mixed Health Behaviours* (15%), and *Multiple Risk Behaviours* (8%) clusters.

Second, to analyse how well the health behaviour clusters reflected self-rated health, we applied an ordinal regression model. Cluster membership probability was analysed with the *Moderately Healthy* as a reference cluster, with adjustments for sociodemographic factors. We also examined the percentage of those who considered their health to be good in each cluster, using cross-tabulation.

Third, we analysed the associations between work-related variables and cluster membership using multinomial regression models. To study the effect of unemployment, we compared the unemployed with all the other participants. When studying other work-related factors, the unemployed were excluded from the analyses. We conducted the analyses one work-related variable at a time, i.e., we did not adjust the work-related variables for each other. We run the analyses with and without adjustments for sociodemographic factors (unadjusted analyses are presented in Appendix 2). The results are presented as odds ratios (OR) and their 95% confidence intervals (CIs). The Bonferroni correction was applied to the overall *p-*values of the work-related variables (eight tests). Participants with missing values in any of the variables used were excluded from the respective analyses. However, we provide the number of missing values for each variable in Tables 1 and 2 detailing the study sample’s characteristics. All the analyses were conducted using the SPSS statistical program (IBM Corp 2021).

## Results

The majority of the participants were women (79%) and the mean age was 43 (Table 1). A quarter had higher than upper secondary education and almost a half reported that they had an insufficient income.

The job industry sector was retail for 42 per cent of the participants, hospitality for 17 per cent, property maintenance for 10 per cent, and 15 per cent indicated it as ‘other’ (Table 2). Most of the participants (89%) were employed and almost half had a permanent, full-time work contract. A third reported working under time pressure very often. Approximately a fifth considered their work to be mentally or physically strenuous. Being completely or moderately dissatisfied with their work was reported by 16% and 47% considered that they were only able to influence their working conditions to some extent or not at all.

The six distinctive clusters *Moderately Healthy, Healthy – Vigorous Exercise, Sedentary Lifestyle, Inadequate Sleep, Mixed Health Behaviours, and Multiple Risk Behaviours* and their characteristics are presented in Table 3.

**Table 3:** Prevalence of the six clusters and distribution of lifestyle characteristics (*n* = 5256)

	Moderately Healthy 28%	Healthy – Vigorous Exercise 19%	Sedentary Lifestyle 16%	Inadequate Sleep 15%	Mixed Health Behaviours 15%	Multiple Risk Behaviours 8%
Leisure-time physical activity (mode)	Moderate exercise	Vigorous Exercise	No leisure-time physical activity	Moderate exercise	Moderate exercise	No leisure-time physical activity
Adequacy of sleep (mode)	Adequate	Adequate	Adequate	Inadequate	Adequate	Inadequate
Smoking status (mode)	Non-smoker	Non-smoker	Non-smoker	Non-smoker	Smoker	Smoker
Heavy drinking occasions per year (mean)	7.3	7.1	7.4	7.6	12.5	60.0
Consumption of fruit, vegetables, and berries per day (mean)	2.5	2.8	2.0	2.2	1.8	1.7

Compared to the *Moderately Healthy* cluster, the members of the *Healthy – Vigorous Exercise* cluster rated their health as better (*p* < .001), whereas the members of the *Mixed Health Behaviours*, *Sedentary Lifestyle*, *Inadequate Sleep*, and *Multiple Risk Behaviours* clusters rated their health as worse (all *p* < .001). The proportion that considered their health to be very good or good was 79% in the *Moderately Healthy* cluster, 88% in the *Healthy – Vigorous Exercise* cluster, 66% in the *Sedentary Lifestyle* cluster, 57% in the *Inadequate Sleep* cluster, 68% in the *Mixed Health Behaviours* cluster, and 49% in the *Multiple Risk Behaviours* cluster.

Results regarding membership in health behaviour clusters differed by industry sector and several work-related factors when the *Moderately Healthy* cluster was treated as the baseline category (Table 4). Those working in the hospitality sector were more likely to be members of the *Mixed Health Behaviours* cluster (OR 1.80, 95% CI 1.15–2.80) and those working in the retail sector were less likely to be members of the *Multiple Risk Behaviours* cluster (OR 0.63, 95% CI 0.40–0.99). Those working very often under time pressure were more likely to be members of the *Inadequate Sleep* (OR 2.62, 95% CI 1.88–3.65), *Mixed Health Behaviours* (OR 1.68, 95% CI 1.21–2.35), or the *Multiple Risk Behaviours* clusters (OR 2.51, 95% CI 1.63–3.85) than of the *Moderately Healthy* cluster. Also, those who reported working under time pressure quite often were more likely to be members of the *Inadequate Sleep* cluster (OR 1.49, 95% CI 1.06–2.11). Those considering their work somewhat or totally mentally strenuous were more likely to be members of the *Inadequate Sleep* (OR 2.28, 95% CI 1.64–3.18 and OR 3.07, 95% CI 2.13–4.43, respectively) or *Multiple Risk Behaviours* (OR 1.71, 95% CI 1.12–2.63 and OR 2.44, 95% CI 1.52–3.90, respectively) clusters. Those who considered their job totally physically strenuous were more likely to be members of the *Inadequate Sleep* (OR 2.07, 95% CI 1.50–2.86) or *Multiple Risk Behaviours* (OR 2.08, 95% CI 1.34–3.22) clusters, and less likely to be members of the *Sedentary Lifestyle* cluster (OR 0.64, 95% CI 0.45–0.91). Finding their job to be somewhat physically strenuous also associated with a lower likelihood of belonging in the *Healthy – Vigorous Exercise* cluster (OR 0.75, 95% CI 0.57–0.98). Workers who were completely or moderately dissatisfied with their jobs more often belonged to the *Healthy – Vigorous Exercise* (OR 1.45, 95% CI 1.03–2.03), *Inadequate Sleep* (OR 3.38, 95% CI 2.43–4.71), and *Multiple Risk Behaviour* (OR 2.77, 95% CI 1.81–4.23) clusters. In addition, those who responded that they are equally satisfied and dissatisfied with their jobs were more likely in the *Inadequate Sleep* cluster (OR 1.82, 95% CI 1.34–2.47).

**Table 4:** Associations found between work-related factors and health behaviour clusters using multinomial regression analyses, each work-related factor as a separate predictor variable in the models and the *Moderately Healthy* cluster as baseline category^§^

Moderately Healthy (ref.)	Healthy –Vigorous Exercise	Sedentary Lifestyle	Inadequate Sleep	Mixed Health Behaviours	Multiple Risk Behaviours			
	95% CI					Un-adjusted *p-*value	Adjusted *p-*value^*^	
Industry sector (*n* = 2450)						0.002	**0.016**	
Other	1.00	1.00	1.00	1.00	1.00			
Retail	0.77 (0.55–1.06)	0.85 (0.60–1.19)	0.94 (0.66–1.34)	0.95 (0.64–1.41)	**0.63** **(0.40–0.99)**			
Hospitality	1.00 (0.68–1.48)	0.94 (0.62–1.42)	1.23 (0.81–1.88)	**1.80** **(1.15–2.80)**	1.17 (0.70–1.95)			
Property maintenance	0.65 (0.41–1.05)	0.75 (0.50–1.21)	1.38 (0.87–2.19)	1.53 (0.93–2.51)	1.01 (0.56–1.81)			
Employment status (*n* = 4736)						0.046	0.368	
Employed/partial pension	1.00	1.00	1.00	1.00	1.00			
Unemployed or laid off	0.86 (0.65–1.15)	0.98 (0.73–1.31)	**0.71** **(0.51–0.98)**	1.12 (0.83–1.50)	1.30 (0.91–1.85)			
Type of employment contract (*n* = 2441)						0.110	0.880	
Fixed-term/part-time	1.00	1.00	1.00	1.00	1.00			
Permanent, full-time	0.85 (0.66–1.10)	1.00 (0.77–1.29)	0.82 (0.63–1.07)	1.23 (0.93–1.61)	1.08 (0.76–1.52)			
Working under time pressure (*n* = 2443)						< 0.001	**< 0.008**	
Sometimes or more seldom	1.00	1.00	1.00	1.00	1.00			
Quite often	1.05 (0.77–1.41)	0.84 (0.62–1.14)	**1.49** **(1.06–2.11)**	1.12 (0.79–1.58)	1.16 (0.72–1.86)			
Very often	1.25 (0.93–1.69)	0.86 (0.63–1.18)	**2.62** **(1.88–3.65)**	**1.68** **(1.21–2.35)**	**2.51** **(1.63–3.85)**			
Mentally strenuous job (*n* = 2418)						< 0.001	**< 0.008**	
Somewhat or fully disagree	1.00	1.00	1.00	1.00	1.00			
Somewhat agree	1.13 (0.85–1.49)	1.08 (0.81–1.44)	**2.28** **(1.64–3.18)**	1.12 (0.82–1.52)	**1.71** **(1.12–2.63)**			
Fully agree	1.11 (0.80–1.55)	1.09 (0.77–1.54)	**3.07** **(2.13–4.43)**	1.23 (0.85–1.77)	**2.44** **(1.52–3.90)**			
Physically strenuous job (*n* = 2441)						< 0.001	**< 0.008**	
Somewhat or fully disagree	1.00	1.00	1.00	1.00	1.00			
Somewhat agree	**0.75** **(0.57–0.98)**	0.86 (0.65–1.13)	1.07 (0.79–1.44)	1.23 (0.90–1.68)	1.33 (0.88–2.00)			
Fully agree	0.75 (0.54–1.04)	**0.64** **(0.45–0.91)**	**2.07** **(1.50–2.86)**	1.39 (0.98–1.99)	**2.08** **(1.34–3.22)**			
Job satisfaction (*n* = 2449)						< 0.001	**< 0.008**	
Completely or moderately satisfied	1.00	1.00	1.00	1.00	1.00			
Equally satisfied and dissatisfied	1.03 (0.77–1.39)	1.13 (0.83–1.53)	**1.82** **(1.34–2.47)**	1.10 (0.79–1.51)	1.50 (0.99–2.26)			
Completely or moderately dissatisfied	**1.45** **(1.03–2.03)**	1.39 (0.97–1.99)	**3.38** **(2.43–4.71)**	1.22 (0.83–1.80)	**2.77** **(1.81–4.23)**			
Able to influence working conditions (*n* = 2408)						0.007	0.056	
A great deal or quite a lot	1.00	1.00	1.00	1.00	1.00			
Somewhat or not at all	0.90 (0.70–1.15)	0.99 (0.77–1.27)	**1.36** **(1.05–1.76)**	0.83 (0.63–1.08)	1.33 (0.94–1.87)			

^§^ Information on employment status was obtained for the whole sample and on other work-related variables only for those who responded to the member survey

^*^ Bonferroni corrected p-value

CI = Confidence interval

Adjusted for sex, age, education level, marital status, and number of children in household

## Discussion

The aim of the study was to investigate how work-related factors are associated with health behaviour clusters among private-sector service workers. We found that those in the retail sector were less likely to be members of the *Multiple Risk Behaviours* cluster and that those working in property maintenance were more likely to be members of the *Mixed Health Behaviours* cluster. Those working under time pressure, doing mentally or physically strenuous work, and feeling dissatisfied with their work were more likely to be members of the *Inadequate Sleep* and *Multiple Risk Behaviours* clusters. Working under time pressure was associated with a higher likelihood of being a member of the *Mixed Health Behaviours* cluster. In addition, feeling dissatisfied with their work but not considering their work physically strenuous indicated a higher likelihood of the worker being a member of the *Healthy – Vigorous Exercise* cluster.

Our research highlights the relationship between several work-related factors and membership of clusters characterized by insufficient sleep or several adverse health behaviours. It is noteworthy that a cluster characterised by inadequate sleep, but otherwise at least moderately healthy lifestyle factors, was identified in addition to the *Multiple Risk Behaviours* cluster and had very similar kind of associations with the unfavourable work-related factors. As previous research has mainly concentrated on work strain and separate health behaviour factors, comparing our results with eclectic associations between work-related factors and health behaviours is challenging. Not only were the clusters data driven but the type of work sector could also influence the examined relations. Among the police force, for example, those with high job strain were more likely to belong to the cluster characterized by low PA level and insufficient FV consumption but also by low-risk drinking (Irizar et al. 2022). In our study, those working in the hospitality branch were more likely to be members of the *Mixed Health Behaviours* cluster, indicating that they were smokers and drank heavily somewhat frequently and consumed FVB less frequently, but also engaged in light PA and slept adequately. A Swedish 27-year cohort study on risky health behaviours including physical inactivity and smoking, using snuff, and drinking alcohol demonstrated that low education level had a strong effect but unemployment or demanding working conditions only had a marginal effect on these health behaviours (Brännlund et al. 2013). Considering the detrimental effects of smoking on health (National Center for Chronic Disease Prevention and Health Promotion (US) Office on Smoking and Health 2014) and the modifiability of working conditions compared to the overall education level, arrangements at workplaces should encourage non-smoking and provide support for smoking cessation.

One of the main findings of our research is that working under time pressure, doing physically and mentally strenuous work, and not being satisfied with one’s job associated with being a member of the cluster characterized by inadequate sleep. Previously, sleep problems have been associated with high job insecurity and low job satisfaction, and their prevalence increased when workers encountered multiple psychosocial work problems (Bertrais et al. 2020). In addition, such stressors as high job demands and job strain have predicted sleep disturbances (Linton et al. 2015). The above-mentioned studies suggest that unfavourable working conditions predict poor sleep, but understandably, insufficient sleep can also reduce a person’s ability to handle the demands of their work by impairing cognitive performance (Van Dongen et al. 2003). Since our study was cross-sectional, we are not able to make assumptions about the direction of the associations. Still, the workplace offers a potential environment to address sleep issues, including sleep inadequacy. A Scandinavian cohort study (Xu et al. 2023) showed that improvements especially in vertical resources such as leadership quality and procedural justice, in addition to collegial support and collaborative culture, reduced persistent sleep disturbances. Also, a work-site intervention improved sleep sufficiency by increasing control over one’s work hours and reducing work-family conflict (Olson et al. 2015), with no decay over time during the 18-month follow-up (Crain et al. 2016). Further research is needed to identify the most effective interventions for addressing the impacts of time pressure and mentally and physically strenuous jobs on sleep.

Our results on the adverse working conditions are consistent in the sense that the same working conditions associated with the *Inadequate Sleep* cluster were related to the cluster characterized by multiple risk behaviours. *Multiple Risk Behaviours* cluster members were also the ones with the poorest self-rated health, followed by *Inadequate Sleep* cluster members. As previous longitudinal studies have shown, for example, constant, high job demands and increased job strains to predict higher BMI (Niskanen et al. 2017) and low control jobs to predict higher mortality rates (Pan et al. 2023), it is justifiable to interpret the results of our study as indicating that unfavourable working conditions do not only associate with several adverse health behaviours but they might ultimately also affect individuals’ health. Preventive measures aiming to diminish both the mental and the physical workload of the workers are beneficial also from the viewpoint of the employer, as it has been suggested that lower quantitative demands and receiving more supervisor recognition, for example, may buffer the effect of sleep problems on sick leaves (Madsen et al. 2016).

In the present study, employment status, type of work contract, and the ability to influence one’s working conditions had no associations with health behaviours. Having a permanent contract has previously been linked to better diet quality (Portero De La Cruz et al. 2021), but we found no differences between health behaviours in terms of whether the work contract was fixed-term/part-time or permanent and full-time. It is possible that union membership, which aims to provide additional financial support for periods of unemployment, acted as a buffer for maintaining habitual health behaviours that demand a certain income level. Service sector work is often characterized by limited ability to influence one’s work pace, space, or the content of tasks, which might explain the lack of discriminating power of these aspects over health behaviours. Still, increasing the opportunities to influence one’s working conditions could be aimed for, as it has been shown to affect several aspects of mental health, especially among young women (Belloni et al. 2022) who form a central group of workers in the service sector.

As several working conditions have been only weakly associated with diet quality (Tanaka et al. 2019), it is possible that relationships are more evident with foods like FVB or fish, often considered expensive – particularly among those with a lower socioeconomic position. In our final cluster solution, we included FVB consumption as an indicator of healthy eating due to FV’s importance for health (Liu 2013; Angelino et al. 2019), and because we found that snacks and sugar-sweetened beverages had less explanatory power in the pre-clustering analyses. A considerable part of the private-sector service workers in the present study perceived their income as inadequate, with (Walsh et al. 2022) showing them having greater odds for food insecurity. Ensuring adequate financial compensation for work is essential for enabling healthy eating. In addition, workplaces should facilitate healthy eating during the working hours, such as through workplace canteen, associated with diets closer to nutrition recommendations (Raulio et al. 2010).

One of the main strengths of the study is its focus on blue- and pink-collar, private-sector service workers with mainly low education and income levels. Thus, this study provides novel knowledge of the hard-to-reach population that is often underrepresented in research. We also examined several working conditions and health behaviours that cluster together. The inclusion of sleep in particular in the health behaviour clusters seems to be important. A major limitation of our study is its low participation rate (6.5%), which restricts the generalizability of our results. Moreover, our sample excluded those who were not members of the working union or those who were not Finnish-speakers. Since they might be even less well-off than the studied sample, this exclusion has possibly made our results more conservative. We assessed the financial situation using self-perceived adequacy of income instead of more objective annual net income, which could be seen as a limitation. Still, subjective evaluation may better capture financial realities, including depths and other expenses, and has, for example, demonstrated stronger associations with self-rated health (Cialani and Mortazavi 2020) and food insecurity (Walsh et al. 2022) than objective income level.

Including factors such as shiftwork or working overtime could have broadened the perspective of our research. Shiftwork is known to have an unfavourable effect on sleep and potentially also on PA and dietary habits (Crowther et al. 2022). Working overtime has been associated, for example, with decreased PA and healthy eating, but not with smoking or alcohol consumption (Taris et al. 2010). Control over one’s work, however, may be more detrimental than the timing of the work as such: unstable and unpredictable work schedules among retail and food service workers have demonstrated stronger associations with poor sleep quality compared to regular night-shift work (Harknett et al. 2020). Our findings among blue- and pink-collar workers add to the existing knowledge by stressing the importance of time pressure, mental and physical strenuosity of the work, and job satisfaction on several health behaviours, including sleep. Given the characteristic on-site nature of service work, employers have a great responsibility in arranging the working conditions to minimise burdens.

## Conclusions

Unfavourable working conditions among private-sector service workers were associated with belonging to clusters characterized by inadequate sleep and several unhealthy behaviours. The private sector should take measures to diminish both the mental and physical workload of service workers, and occupational health services should better recognize multiple health risk behaviours, including poor sleep. As private-sector service workers have relatively low education and income levels, their opportunities to freely choose their career paths on the labour market or to arrange their private lives to support adequate recovery might be more limited and therefore they are much more dependent on the support they receive from their workplace.

### Electronic supplementary material

Below is the link to the electronic supplementary material.

Supplementary Material 1

Supplementary Material 2

## Data Availability

Data will be made available upon reasonable request.
