# Supplementary material for: Association between work-related factors and health behaviour clusters among Finnish private-sector service workers
Source: Int Arch Occup Environ Health. 2024 May 7;97(6):641–50. doi: 10.1007/s00420-024-02069-9 (PMC11245410; doi:10.1007/s00420-024-02069-9)
Supplement: Supplementary file 1 — Supplementary Material 1 [file 420_2024_2069_MOESM1_ESM.pdf]

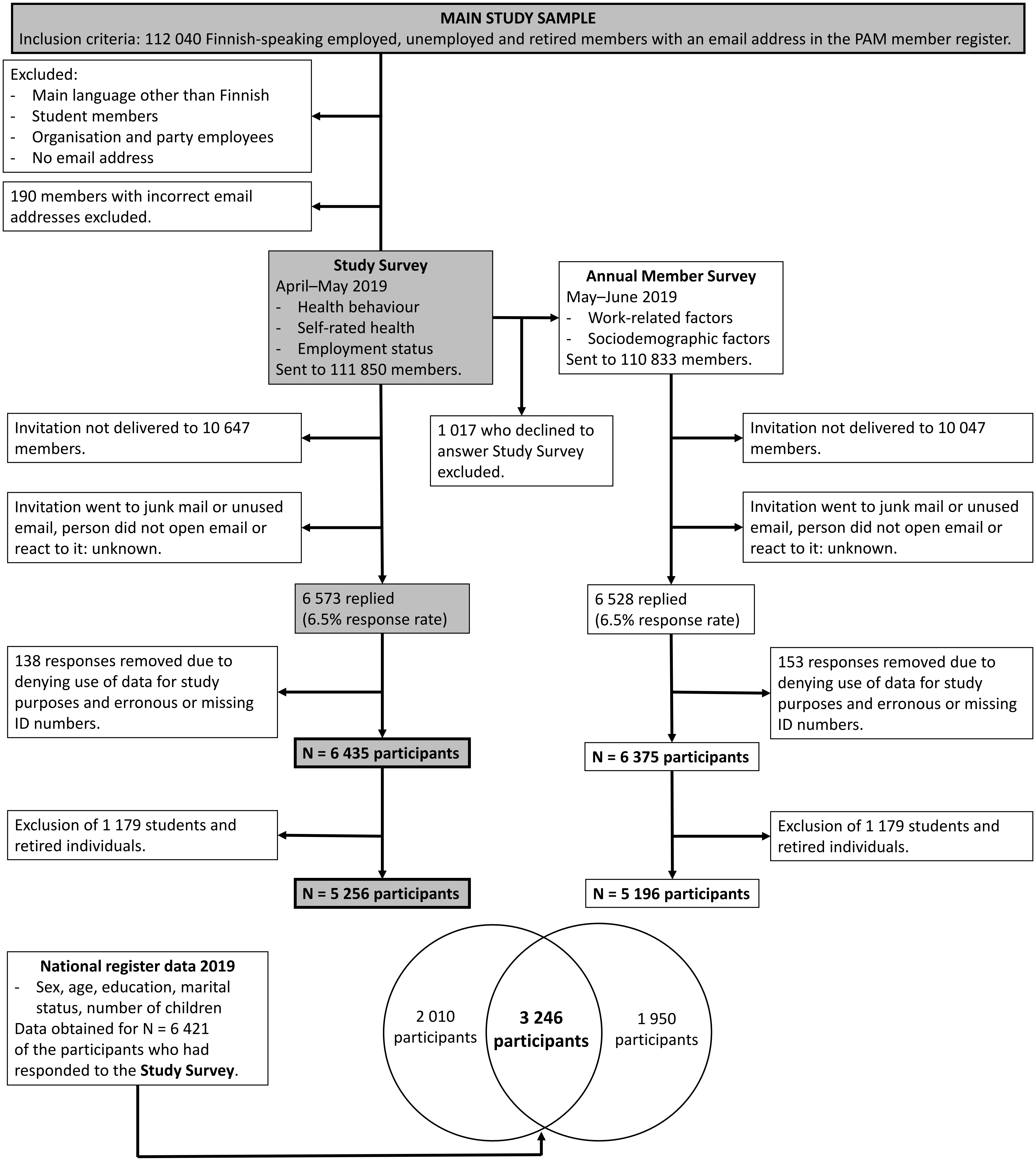

. Appendix 1. The study sample, inclusion and exclusion criteria, and participation in the PAMEL study.
