# Supplementary material for: Association between work-related factors and health behaviour clusters among Finnish private-sector service workers
Source: Int Arch Occup Environ Health. 2024 May 7;97(6):641–50. doi: 10.1007/s00420-024-02069-9 (PMC11245410; doi:10.1007/s00420-024-02069-9)
Supplement: Supplementary file 2 — Supplementary Material 2 [file 420_2024_2069_MOESM2_ESM.docx]

Appendix 2. Unadjusted associations found between work-related factors and health behaviour clusters using multinomial regression analyses, each work-related factor as a separate predictor variable in the models and the *Moderately Healthy* cluster as baseline category^§^.

| *Moderately Healthy* (ref.) | *Healthy* –*Vigorous Exercise* | *Sedentary*  *Lifestyle* | *Inadequate Sleep* | *Mixed Health*  *Behaviours* | *Multiple Risk*  *Behaviours* |  |  |  |
| --- | --- | --- | --- | --- | --- | --- | --- | --- |
|  | 95% CI | | | | | Un-adjusted *p-*value | Adjusted  *p-*value^*^ | |
| Industry sector (n=2676) |  |  |  |  |  | <0.001 | **<0.008** |  |
| Other | 1.00 | 1.00 | 1.00 | 1.00 | 1.00 |  |  |  |
| Retail | **0.70**  **(0.51–0.95)** | 0.80  (0.57–1.10) | 0.92  (0.65–1.30) | 0.95  (0.66–1.37) | **0.65**  **(0.42–1.00)** |  |  |  |
| Hospitality | 0.96  (0.66–1.38) | 0.92  (0.62–1.37) | 1.17  (0.78–1.76) | **1.62**  **(1.07–2.46)** | 1.11  (0.68–1.82) |  |  |  |
| Property maintenance | **0.61**  **(0.39–0.95)** | 0.74  (0.47–1.17) | 1.36  (0.88–2.11) | 1.51  (0.95–2.40) | 1.05  (0.60–1.82) |  |  |  |
| Employment status (n=5256) |  |  |  |  |  | <0.001 | **<0.008** |  |
| Employed/partial pension | 1.00 | 1.00 | 1.00 | 1.00 | 1.00 |  |  |  |
| Unemployed or laid off | **0.75**  **(0.58–0.99)** | 0.89  (0.68–1.17) | **0.71**  **(0.53–0.95)** | 1.16  (0.90–1.51) | **1.40**  **(1.03–1.90)** |  |  |  |
| Type of employment contract (n=2666) |  |  |  |  |  | 0.062 | 0.496 |  |
| Fixed-term/part-time | 1.00 | 1.00 | 1.00 | 1.00 | 1.00 |  |  |  |
| Permanent, full-time | 0.87  (0.69–1.10) | 1.06  (0.83–1.35) | 0.88  (0.69–1.12) | **1.28**  **(1.00–1.65)** | 1.04  (0.76–1.42) |  |  |  |
| Working under time pressure (n=2669) |  |  |  |  |  | <0.001 | **<0.008** |  |
| Sometimes or more seldom | 1.00 | 1.00 | 1.00 | 1.00 | 1.00 |  |  |  |
| Quite often | 1.05  (0.77–1.41) | 0.80  (0.60–1.07) | **1.44**  **(1.04–2.01)** | 1.11  (0.80–1.53) | 1.10  (0.71–1.70) |  |  |  |
| Very often | 1.16  (0.88–1.55) | 0.85  (0.64–1.15) | **2.60**  **(1.89–3.58)** | **1.71**  **(1.25–2.34)** | **2.25**  **(1.51–3.36)** |  |  |  |
| Mentally strenuous job (n=2640) |  |  |  |  |  | <0.001 | **<0.008** |  |
| Somewhat or fully disagree | 1.00 | 1.00 | 1.00 | 1.00 | 1.00 |  |  |  |
| Somewhat agree | 1.12  (0.86–1.46) | 1.00  (0.76–1.31) | **2.24**  **(1.63–3.08)** | 1.05  (0.79–1.40) | **1.74**  **(1.16–2.61)** |  |  |  |
| Fully agree | 1.09  (0.79–1.49) | 1.01  (0.73–1.40) | **3.03**  **(2.14–4.31)** | 1.17  (0.83–1.64) | **2.49**  **(1.60–3.86)** |  |  |  |
| Physically strenuous job (n=2665) |  |  |  |  |  | <0.001 | **<0.008** |  |
| Somewhat or fully disagree | 1.00 | 1.00 | 1.00 | 1.00 | 1.00 |  |  |  |
| Somewhat agree | **0.77**  **(0.60–1.00)** | 0.92  (0.71–1.20) | 1.09  (0.81–1.45) | 1.32  (0.99–1.88) | 1.31  (0.90–1.90) |  |  |  |
| Fully agree | **0.71**  **(0.52–0.97)** | **0.63**  **(0.45–0.90)** | **2.03**  **(1.50–2.76)** | 1.35  (0.97–1.88) | **1.90**  **(1.27–2.86)** |  |  |  |
| Job satisfaction (n=2675) |  |  |  |  |  | <0.001 | **<0.008** |  |
| Completely or moderately satisfied | 1.00 | 1.00 | 1.00 | 1.00 | 1.00 |  |  |  |
| Equally satisfied and dissatisfied | 0.94  (0.71–1.26) | 1.13  (0.84–1.50) | **1.79**  **(1.33–2.40)** | 1.12  (0.83–1.51) | **1.63**  **(1.11–2.39)** |  |  |  |
| Completely or moderately dissatisfied | **1.47**  **(1.07–2.03)** | 1.37  (0.97–1.94) | **3.61**  **(2.63–4.95)** | 1.28  (0.89–1.84) | **3.35**  **(2.26–4.98)** |  |  |  |
| Able to influence working conditions (n=2630) |  |  |  |  |  | <0.001 | **<0.008** |  |
| A great deal or quite a lot | 1.00 | 1.00 | 1.00 | 1.00 | 1.00 |  |  |  |
| Somewhat or not at all | 0.81  (0.64–1.02) | 0.95  (0.75–1.22) | **1.35**  **(1.05–1.73)** | 0.83  (0.65–1.07) | 1.24  (0.90–1.71) |  |  |  |

^§^ Information on employment status was obtained for the whole sample and on other work-related variables only for those who responded to the member survey.

^*^ Bonferroni corrected p-value.

CI=Confidence interval
